# COSMIN guideline for systematic reviews of patient-reported outcome measures

**DOI:** 10.1007/s11136-018-1798-3

**Published:** 2018-02-12

**Authors:** C. A. C. Prinsen, L. B. Mokkink, L. M. Bouter, J. Alonso, D. L. Patrick, H. C. W. de Vet, C. B. Terwee

**Affiliations:** 1 Department of Epidemiology and Biostatistics, Amsterdam Public Health Research Institute, VU University Medical Center, De Boelelaan 1089a, 1081 HV Amsterdam, The Netherlands; 2 Health Services Research Unit, IMIM-Hospital del Mar Medical Research Institute; CIBER Epidemiología y Salud Pública (CIBERESP), Barcelona, Spain; 3 Department of Health Services, University of Washington, Seattle, WA USA; 4 Department of Epidemiology and Biostatistics, Amsterdam Public Health Research Institute, VU University Medical Center, P.O. Box 7057, 1007 MB Amsterdam, The Netherlands

**Keywords:** COSMIN, Systematic review, Measurement properties, PROM, Outcome measurement instrument, Outcome measures, Methodology

## Abstract

**Purpose:**

Systematic reviews of patient-reported outcome measures (PROMs) differ from reviews of interventions and diagnostic test accuracy studies and are complex. In fact, conducting a review of one or more PROMs comprises of multiple reviews (i.e., one review for each measurement property of each PROM). In the absence of guidance specifically designed for reviews on measurement properties, our aim was to develop a guideline for conducting systematic reviews of PROMs.

**Methods:**

Based on literature reviews and expert opinions, and in concordance with existing guidelines, the COnsensus-based Standards for the selection of health Measurement INstruments (COSMIN) steering committee developed a guideline for systematic reviews of PROMs.

**Results:**

A consecutive ten-step procedure for conducting a systematic review of PROMs is proposed. Steps 1–4 concern preparing and performing the literature search, and selecting relevant studies. Steps 5–8 concern the evaluation of the quality of the eligible studies, the measurement properties, and the interpretability and feasibility aspects. Steps 9 and 10 concern formulating recommendations and reporting the systematic review.

**Conclusions:**

The COSMIN guideline for systematic reviews of PROMs includes methodology to combine the methodological quality of studies on measurement properties with the quality of the PROM itself (i.e., its measurement properties). This enables reviewers to draw transparent conclusions and making evidence-based recommendations on the quality of PROMs, and supports the evidence-based selection of PROMs for use in research and in clinical practice.

## Introduction

A patient-reported outcome (PRO) is any aspect of a patient’s health status that is directly assessed by the patient without the interpretation of the patient’s response by anyone other than the patient [1]. PROs are most commonly assessed by means of self-administered questionnaires, also known as patient-reported outcome measures (PROMs). It is known, however, that the quality of PROMs used varies considerably, and it is usually not apparent weather the most reliable and valid PROM has been selected [2–5].

Systematic reviews of PROMs are important tools for selecting the most suitable PROM to measure a construct of interest in a specific study population. High quality systematic reviews can provide a comprehensive overview of the measurement properties of PROMs and supports evidence-based recommendations in the selection of the most suitable PROM for a given purpose (i.e., research or clinical practice, or discriminative, evaluative or predictive applications). Different PROMs may be suitable for different purposes and may depend on feasibility aspects as well. Systematic reviews of PROMs can also identify gaps in knowledge about the measurement properties of the PROMs at issue, which can be used to design new studies on measurement properties.

The number of systematic reviews of PROMs has increased from hardly one per year in the beginning of the 1990s to more than 100 each year currently [6]. A recent review of the quality of systematic reviews of health-related outcome measurement instruments showed that there is considerable room for improvement [7].

The COnsensus-based Standards for the selection of health Measurement INstruments (COSMIN) initiative aims to facilitate the selection of high quality PROMs for research and clinical practice. One of the tools developed is a protocol for systematic reviews of PROMs that was available on the COSMIN website since 2011 (http://www.cosmin.nl) [8]. In the absence of an extensive and published guideline for systematic reviews of PROMs, the COSMIN steering committee (i.e., the authors of this paper) aimed to extend this protocol into a comprehensive methodological guideline for systematic reviews of PROMs. In ten consecutive steps, the present guideline describes the methodology of systematic reviews of existing PROMs, for which at least some information on its measurement properties is available, and that are used for evaluative purposes, and will support the selection of PROMs for a specific purpose. Detailed information supporting the conduct of a systematic review can be found in the accompanying “COSMIN methodology for systematic reviews of PROMs—user manual”, as well as in the “COSMIN methodology for assessing the content validity of PROMs—user manual”, available on the COSMIN website [8–10]. These user manuals are supporting documents to the present guideline and intended to support systematic reviewers in conducting systematic reviews of PROMs. The “COSMIN methodology for systematic reviews of PROMs—user manual” provides detailed information for each particular step of a systematic review of PROMs, supported by multiple examples for different scenario’s.

## Methods

In the absence of empirical evidence, the present COSMIN guideline for systematic reviews of PROMs is based on our experience that we (that is: the COSMIN steering committee) have gained over the past years in conducting systematic reviews of PROMs [11, 12], in supporting other systematic reviewers in their work [13, 14], and in the development of COSMIN methodology [15, 16]. In addition, we have studied the quality of systematic reviews of PROMs in two consecutive reviews [7, 17], and in reviews that have used the COSMIN methodology we have specifically searched for the comments made by review authors relating to the COSMIN methodology. Further, we have had iterative discussions by the COSMIN steering committee, both at face-to-face meetings (CP, WM, HdV and CT) and by email. We gained experience from results of a recent Delphi study on the content validity of PROMs [18], and from results of a previous Delphi study on the selection of outcome measurement instruments for outcomes included in core outcome sets (COS) [19]. Further, the guideline was developed in concordance with existing guidelines for reviews, such as the Cochrane handbook for systematic reviews of interventions [20] and for diagnostic test accuracy reviews [21], the PRISMA Statement [22], the Institute of Medicine standards for systematic reviews of comparative effectiveness research [23], and the Grading of Recommendations Assessment, Development and Evaluation (GRADE) principles [24].

## Results

**Fig. 1:**
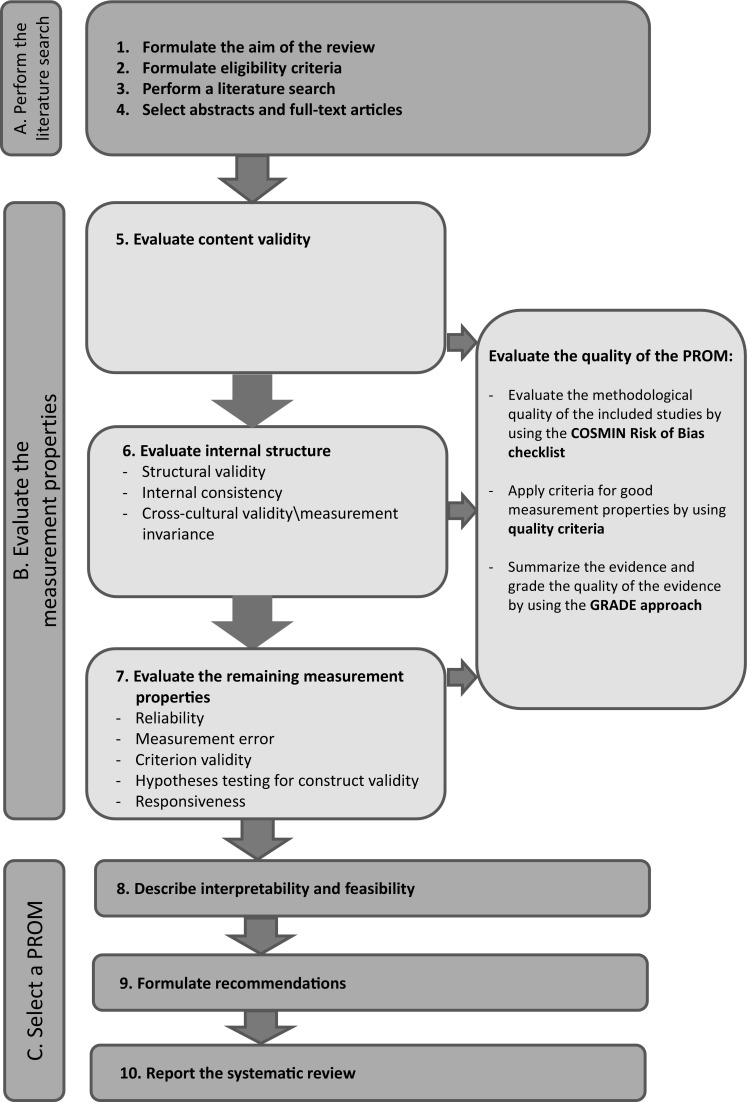
Ten steps for conducting a systematic review of PROMs

A consecutive ten-step procedure for conducting a systematic review of PROMs is recommended (Fig. 1). These steps are subdivided in three parts: A, B, and C.

### Part A. Perform the literature search

Part A consists of steps 1–4 and generally, these steps are standard procedures when performing systematic reviews, and are in agreement with existing guidelines for reviews [20, 21].

#### Step 1. Formulate the aim of the review

The aim of a systematic review of PROMs focuses on the quality of the PROMs. It should include the following four key elements: (1) the construct; (2) the population(s); (3) the type of instrument(s); and (4) the measurement properties of interest. For example: “our aim is to critically appraise, compare and summarize the quality of the measurement properties of all self-report fatigue questionnaires for patients with multiple sclerosis (MS), Parkinson’s disease (PD) or stroke” [25].

#### Step 2. Formulate eligibility criteria

The eligibility criteria should be in agreement with the four key elements of the review aim: (1) the PROM(s) should aim to measure the construct of interest; (2) the study sample (e.g., or an arbitrary majority, e.g., ≥ 50%) should represent the population of interest; (3) the study should concern PROMs; and (4) the aim of the study should be the evaluation of one or more measurement properties, the development of a PROM (to rate the content validity), or the evaluation of the interpretability of the PROMs of interest (e.g., evaluating the distribution of scores in the study population, percentage of missing items, floor and ceiling effects, the availability of scores and change scores for relevant (sub)groups, and the minimal important change (MIC) or minimal important difference [26]). We recommend to exclude studies that only use the PROM as an outcome measurement instrument. These studies provide indirect evidence on the measurement properties of the PROM. This concerns, for instance, studies in which the PROM is used to measure the outcome (e.g., in randomized controlled trials), or studies in which the PROM is used in a validation study of another instrument. We further recommend to include only full-text articles, because, often, very limited information on the design of a study is found in abstracts, which will hamper the quality assessment of the study and the results of the measurement properties in steps 5–7.

#### Step 3. Perform a literature search

In agreement with the Cochrane methodology [20, 21], and based on consensus [19], MEDLINE and EMBASE are considered to be the minimum databases to be searched. In addition, it is recommended to search in other (content-specific) databases, depending on the construct and population of interest, for example Web of Science, Scopus, CINAHL, or PsycINFO.

An adequate search strategy consists of a comprehensive collection of search terms (i.e., index terms and free text words) for the four key elements of the review aim: (1) construct; (2) population(s); (3) type of instrument(s); and (4) measurement properties. It is recommended to consult a clinical librarian as well as experts on the construct and study population of interest.

A comprehensive PROM filter has been developed for PubMed by the Patient-Reported Outcomes Measurement Group, University of Oxford, that can be used as a search block for type of measurement instrument(s), and is available on the COSMIN website [8]. Regarding search terms for measurement properties we recommend to use a highly sensitive validated search filter for finding studies on measurement properties [27], which is available for PubMed and EMBASE, which can be found on the COSMIN website [8]. An example of a PubMed search strategy can be found in the COSMIN user manual [9].

In agreement with the Cochrane methodology, it is recommended to search databases from the date of inception until present [20, 21]. The use of language restrictions depends on the inclusion criteria defined in step 2. In general, it is recommend not to use language restrictions in the search strategy, even if there are no resources to translate the articles for the review. In this way, review authors are at least able to report their existence.

#### Step 4. Select abstracts and full-text articles

It is generally recommended to perform the selection of abstracts and full-text articles by two reviewers independently [20, 21]. If a study seems relevant by at least one reviewer based on the abstract, or in case of doubt, the full-text article needs to be retrieved and screened. Differences should be discussed and if consensus between the two reviewers cannot be reached, it is recommended to consult a third reviewer. It is also recommended to check all references of the included articles to search for additional potentially relevant studies. If many new articles are found, the initial search strategy might have been insufficiently comprehensive and may need to be improved and redone.

### Part B. Evaluate the measurement properties

Part B consists of steps 5–7 and concerns the evaluation of the measurement properties of the included PROMs, and consists of three sub-steps (Fig. 1). First, the methodological quality of each single study on a measurement property is assessed using the COSMIN Risk of Bias checklist [28]. Each study is rated as either very good, adequate, doubtful or inadequate quality. Second, the result of each single study on a measurement property is rated against the updated criteria for good measurement properties [29] on which consensus was achieved [19] and slightly modified based on recent new insights (Table 1). Each result is rated as either sufficient (+), insufficient (−), or indeterminate (?). Third, the evidence will be summarized and the quality of the evidence will be graded by using the GRADE approach. The results of all available studies on a measurement property are quantitatively pooled or qualitatively summarized and compared against the criteria for good measurement properties to determine whether—overall—the measurement property of the PROM is sufficient (+), insufficient (−), inconsistent (±), or indeterminate (?). The focus is here on the PROM, while in the previous sub-steps the focus was on the single studies. If the ratings per study are all sufficient (or all insufficient), the results can be statistically pooled and the overall rating will be sufficient (+) (or insufficient (−)), based on the criteria of good measurement properties. If the results are inconsistent, explanations for inconsistency (e.g., different study populations or methods) should be explored. If an explanation is found, overall ratings should be provided for relevant subgroups with consistent results (e.g., adults versus children, patients with acute versus chronic disease, different (language) versions of a PROM, etc.). If no explanation is found, the overall rating will be inconsistent (±). If not enough information is available, the overall rating will be indeterminate (?). In the COSMIN user manual, detailed information can be found on how the pooled or summarized results on a measurement property can be rated against the criteria for good measurement properties [9].

**Table 1:** Updated criteria for good measurement properties

Measurement property	Rating	Criteria
Structural validity	+	**CTT** CFA: CFI or TLI or comparable measure > 0.95 OR RMSEA < 0.06 OR SRMR < 0.08^a^
		**IRT/Rasch** No violation of unidimensionality^b^: CFI or TLI or comparable measure > 0.95 OR RMSEA < 0.06 OR SRMR < 0.08 *AND* no violation of local independence: residual correlations among the items after controlling for the dominant factor < 0.20 OR Q3’s < 0.37 *AND* no violation of monotonicity: adequate looking graphs OR item scalability > 0.30 *AND* adequate model fit IRT: χ^2^ > 0.001 Rasch: infit and outfit mean squares ≥ 0.5 and ≤ 1.5 OR Z-standardized values > −2 and < 2
	?	CTT: not all information for ‘+’ reported IRT/Rasch: model fit not reported
	−	Criteria for ‘+’ not met
Internal consistency	+	At least low evidence^c^ for sufficient structural validity^d^ AND Cronbach’s alpha(s) ≥ 0.70 for each unidimensional scale or subscale^e^
	?	Criteria for “At least low evidence^c^ for sufficient structural validity^d^” not met
	−	At least low evidence^c^ for sufficient structural validity^d^ AND Cronbach’s alpha(s) < 0.70 for each unidimensional scale or subscale^e^
Reliability	+	ICC or weighted Kappa ≥ 0.70
	?	ICC or weighted Kappa not reported
	−	ICC or weighted Kappa < 0.70
Measurement error	+	SDC or LoA < MIC^d^
	?	MIC not defined
	−	SDC or LoA > MIC^d^
Hypotheses testing for construct validity	+	The result is in accordance with the hypothesis^f^
	?	No hypothesis defined (by the review team)
	−	The result is not in accordance with the hypothesis^f^
Cross-cultural validity\measurement invariance	+	No important differences found between group factors (such as age, gender, language) in multiple group factor analysis OR no important DIF for group factors (McFadden’s R^2^ < 0.02)
	?	No multiple group factor analysis OR DIF analysis performed
	−	Important differences between group factors OR DIF was found
Criterion validity	+	Correlation with gold standard ≥ 0.70 OR AUC ≥ 0.70
	?	Not all information for ‘+’ reported
	−	Correlation with gold standard < 0.70 OR AUC < 0.70
Responsiveness	+	The result is in accordance with the hypothesis^f^ OR AUC ≥ 0.70
	?	No hypothesis defined (by the review team)
	−	The result is not in accordance with the hypothesis^f^ OR AUC < 0.70

The criteria are based on, e.g., Terwee et al. [29] and Prinsen et al. [19]

*AUC* area under the curve, *CFA* confirmatory factor analysis, *CFI* comparative fit index, *CTT* classical test theory, *DIF* differential item functioning, *ICC* intraclass correlation coefficient, *IRT* item response theory, *LoA* limits of agreement, *MIC* minimal important change, *RMSEA* root mean square error of approximation, *SEM* standard error of measurement, *SDC* smallest detectable change, *SRMR* standardized root mean residuals, *TLI* Tucker–Lewis index

“+” = sufficient, “−” = insufficient, “?” = indeterminate

^a^To rate the quality of the summary score, the factor structures should be equal across studies

^b^Unidimensionality refers to a factor analysis per subscale, while structural validity refers to a factor analysis of a (multidimensional) patient-reported outcome measure

^c^As defined by grading the evidence according to the GRADE approach

^d^This evidence may come from different studies

^e^The criteria ‘Cronbach alpha < 0.95’ was deleted, as this is relevant in the development phase of a PROM and not when evaluating an existing PROM

^f^The results of all studies should be taken together and it should then be decided if 75% of the results are in accordance with the hypotheses

The overall ratings of each measurement property [i.e., sufficient (+), insufficient (–), inconsistent (±)] will be accompanied by a grading for the quality of the evidence. This indicates how confident we are that the pooled results or overall ratings are trustworthy. Note that in case the overall rating for a specific measurement property will be indeterminate (?) one will not be able to judge the quality of the PROMs, so there will be no grading of the quality of the evidence. The GRADE approach for systematic reviews of intervention studies specifies four levels of quality evidence (i.e., high, moderate, low, or very low quality evidence), depending on the presence of five factors: risk of bias, indirectness, inconsistency, imprecision, and publication bias [24]. Here, we introduce a modified GRADE approach for grading the quality of the evidence in systematic reviews of PROMs. The GRADE approach is used to downgrade the quality of evidence when there are concerns about the trustworthiness of the results. Four of the five GRADE factors have been adopted in the COSMIN methodology: risk of bias (i.e., the methodological quality of the studies), inconsistency (i.e., unexplained inconsistency of results across studies), imprecision (i.e., total sample size of the available studies), and indirectness (i.e., evidence from different populations than the population of interest in the review) (Table 2). The quality of the evidence is graded for each measurement property and for each PROM separately. The starting point is always the assumption that the pooled or overall result is of high quality. The quality of evidence is subsequently downgraded by one or two levels per factor to moderate, low, or very low (for definitions, see Table 3) when there is risk of bias, (unexplained) inconsistency, imprecision, or indirect results. Specific details on how to down grade are explained in the COSMIN user manual [9]. We recommend that quality assessment is done by two reviewers independently and that consensus among the reviewers is reached, if necessary with help of a third reviewer.

**Table 2:** Modified GRADE approach for grading the quality of evidence

Quality of evidence	Lower if
High	Risk of bias −1 Serious −2 Very serious −3 Extremely serious Inconsistency −1 Serious −2 Very serious Imprecision −1 total *n* = 50–100 −2 total *n* < 50 Indirectness −1 Serious −2 Very serious
Moderate	
Low	
Very low	

The starting point is the assumption that the evidence is of high quality. The quality of evidence is subsequently downgraded with one or two levels for each factor (i.e., risk of bias, inconsistency, imprecision, indirectness) to moderate, low, or very low when there is risk of bias (low study quality), (unexplained) inconsistency in results, or indirect results [44]. Information on how to downgrade is described in detail in the COSMIN user manual [9]

*n* = sample size

**Table 3:** Definitions of quality levels

Quality level	Definition
High	We are very confident that the true measurement property lies close to that of the estimate of the measurement property
Moderate	We are moderately confident in the measurement property estimate: the true measurement property is likely to be close to the estimate of the measurement property, but there is a possibility that it is substantially different
Low	Our confidence in the measurement property estimate is limited: the true measurement property may be substantially different from the estimate of the measurement property
Very low	We have very little confidence in the measurement property estimate: the true measurement property is likely to be substantially different from the estimate of the measurement property

These definitions were adapted from the GRADE approach [24]. Information on how to downgrade is described in detail in the COSMIN user manual [9]

Note that each version of the PROM should be considered separately in the review (i.e., different versions for subgroups of patients, different language versions, etc.).

#### Step 5. Evaluate content validity

Content validity refers to the degree to which the content of a PROM is an adequate reflection of the construct to be measured [30]. Content validity is considered to be the most important measurement property, because it should be clear that the items of the PROM are relevant, comprehensive, and comprehensible with respect to the construct of interest and study population. The evaluation of content validity requires a subjective judgment by the reviewers. In this judgement, the PROM development study, the quality and results of additional content validity studies on the PROMs (if available), and a subjective rating of the content of the PROMs by the reviewers is taken into account. Guidance on how to evaluate the content validity of PROMs can be found elsewhere [10].

If there is high quality evidence that the content validity of a PROM is insufficient, the PROM will not be further considered in steps 6–8 of the systematic review and one can directly draw a recommendation for this PROM in step 9.

#### Step 6. Evaluate internal structure

The internal structure refers to how the different items in the PROM are related, which is important to know for deciding how items might be combined into a scale or subscale. This step concerns an evaluation of structural validity (including unidimensionality), internal consistency, and cross-cultural validity and other forms of measurement invariance. Here we are referring to testing of existing PROMs; not further refinement or development of new PROMs. These three measurement properties focus on the quality of the individual items and the relationships between the items in contrast to the remaining measurement properties at step 7. We recommend to evaluate these measurement properties directly after evaluating the content validity of a PROM. As evidence for structural validity (or unidimensionality) of a scale or subscale is a prerequisite for the interpretation of internal consistency analyses (i.e., Cronbach’s alpha’s), we recommend to first evaluate structural validity (step 6.1), to be followed by internal consistency (step 6.2) and cross-cultural validity\measurement invariance (step 6.3).

Step 6 is only relevant for PROMs that are based on a reflective model that assumes that all items in a scale or subscale are manifestations of one underlying construct and are expected to be correlated. An example of a reflective model is the measurement of anxiety; anxiety manifests itself in specific characteristics, such as worrying thoughts, panic, and restlessness. By asking patients about these characteristics, we can assess the degree of anxiety (i.e., the items are a reflection of the construct) [31]. If the items in a scale or subscale are not supposed to be correlated (i.e., a formative model), these analyses are not relevant and step 6 can be omitted. If it is not reported whether a PROM is based on a reflective or formative model, the reviewers need to decide on the content of the PROM whether it is likely based on a reflective or a formative model [32].

##### Step 6.1. Evaluate structural validity

Structural validity refers to the degree to which the scores of a PROM are an adequate reflection of the dimensionality of the construct to be measured [30] and is usually assessed by factor analysis or IRT/Rasch analysis. In a systematic review, it is helpful to make a distinction between studies where factor analysis is performed to assess structural validity, or to assess the unidimensionality of each subscale separately/per subscale. To assess structural validity, FA is performed on all items of a PROM to evaluate the (hypothesized) number of subscales of the PROM and the clustering of items within subscales (i.e., structural validity studies). To assess unidimentionality per subscale, multiple factor analyses are performed on the items of each subscale separately to assess whether each subscale on its own measures a single construct (i.e., unidimensionality studies). These analyses are sufficient for the interpretation of internal consistency analyses (step 6.2) and for IRT/Rasch analysis, but it does not provide evidence for structural validity as part of construct validity.

The evaluation of structural validity consists of the three sub-steps that are described under Part B: (1) the evaluation of the methodological quality of the included studies; (2) applying criteria for good measurement properties; and (3) summarizing the evidence and grading the quality of the evidence.

If there is high quality evidence that the structural validity of a PROM is insufficient, one should reconsider further evaluation of this PROM in the subsequent steps.

##### Step 6.2. Evaluate internal consistency

Internal consistency refers to the degree of interrelatedness among the items and is often assessed by Cronbach’s alpha [30, 33]. Similar to the evaluation of structural validity, the evaluation of internal consistency also consists of three sub-steps, as described above.

##### Step 6.3. Evaluate cross-cultural validity\measurement invariance

Cross-cultural validity\measurement invariance refers to the degree to which the performance of the items on a translated or culturally adapted PROM are an adequate reflection of the performance of the items of the original version of the PROM [30]. Cross-cultural validity\measurement invariance should be evaluated when a PROM is or will be used in different ‘cultural’ populations, i.e., populations that differ in ethnicity, language, gender, or age groups, but also different patient populations are considered here [9]. Cross-cultural validity\measurement invariance is evaluated by assessing whether differential item functioning (DIF) occurs using, e.g., logistic regression analyses, or whether factor structure and factor loadings are equivalent across groups using multigroup confirmatory factor analysis (MGCFA). Measurement invariance and non-DIF refer to whether respondents from different groups with the same latent trait level (allowing for group differences) respond similarly to a particular item [34]. The evaluation of cross-cultural validity\measurement invariance also consists of the three sub-steps described above.

#### Step 7. Evaluate the remaining measurement properties

Subsequently, the remaining measurement properties (reliability, measurement error, criterion validity, hypotheses testing for construct validity, and responsiveness) should be evaluated, again following the three sub-steps described above. Unlike content validity and internal structure, the evaluation of these measurement properties provides information on the quality of the scale or subscale as a whole, rather than on item level.

In the evaluation of the measurement properties of the included PROMs, there are a few important issues that should be taken into consideration. For applying the criteria for good measurement error, information is needed on the smallest detectable change (SDC) or limits of agreement (LoA), as well as on the MIC. This information may come from different studies. The MIC should have been determined using an anchor-based longitudinal approach [35–38]. The MIC is best calculated from multiple studies and by using multiple anchors [39, 40]. If not enough information is available to judge whether the SDC or LoA is smaller than the MIC, we recommend to just report the information that is available on the SDC or LoA without grading the quality of evidence (note that information on the MIC alone provides information on the interpretability of a PROM).

With regard to hypotheses testing for construct validity and responsiveness, it is recommended for reviewers to formulate hypotheses themselves to evaluate the results against [9, 28]. These hypotheses are formulated in line with the review aim and include expected relationships, for example, between the PROM(s) under review and the comparison instrument(s) that is/are used to compare the PROM(s) against, and the expected direction and magnitude of the correlation. Examples of generic hypotheses can be found in Table 4. In this way, all results found in the included studies can be compared against the same set of hypotheses. When at least 75% of the results are in accordance with the hypotheses, the summary result is rated as ‘sufficient’. Herewith, more robust conclusions can be drawn about the construct validity of the PROM.

**Table 4:** Generic hypotheses to evaluate construct validity and responsiveness

Generic hypotheses	
1	Correlations with (changes in) instruments measuring similar constructs should be ≥ 0.50
2	Correlations with (changes in) instruments measuring related, but dissimilar constructs should be lower, i.e., 0.30–0.50
3	Correlations with (changes in) instruments measuring unrelated constructs should be < 0.30
4	Correlations with (changes in) instruments measuring similar constructs should differ by a minimum of 0.10 from correlations with (changes in) instruments measuring related but dissimilar constructs Correlations with (changes in) instruments measuring related but dissimilar constructs should differ by a minimum of 0.10 from correlations with (changes in) instruments measuring unrelated constructs
5	Meaningful changes between relevant (sub)groups (e.g., patients with expected high versus low levels of the construct of interest)
6	For responsiveness, AUC should be ≥ 0.70

*AUC* area under the curve with an external measure of change used as the ‘gold standard’

### Part C. Select a PROM

Part C consists of steps 8–10 and concerns the evaluation of the interpretability and feasibility of PROMs, formulating recommendations, and reporting the systematic review.

#### Step 8. Describe interpretability and feasibility

Interpretability is defined as the degree to which one can assign qualitative meaning (that is, clinical or commonly understood connotations) to a PROM’s quantitative scores or change in scores [30]. For example, information on the distribution of scores is needed to interpret some measurement properties, it may reveal clustering of scores and indicates whether this is causing floor and ceiling effects [31]. Feasibility is defined as the ease of application of the PROM in its intended setting, given constraints such as time or money [41]. It refers to aspects such as completion time, cost of an instrument, length of the instrument, type and ease of administration, etc. [19]. Feasibility applies to patients completing the PROM (self-administered) and researchers or clinicians who interview or hand over the PROM to patients. Interpretability and feasibility are not measurement properties, because they do not refer to the quality of a PROM. However, they are considered important aspects for a well-considered selection of a PROM. In case there are two PROMs that are very difficult to differentiate in terms of quality, it is recommended that feasibility aspects should be taken into consideration in the selection of the most appropriate instrument. Reviewers should decide what is feasible in their time frame and within their budget [19].

#### Step 9. Formulate recommendations

Recommendations on the most suitable PROM for use in an evaluative application are formulated with respect to the construct of interest and study population. To come to an evidence-based and fully transparent recommendation [31], we recommend to categorize the included PROMs into three categories: (A) PROMs that have potential to be recommended as the most suitable PROM for the construct and population of interest (i.e., PROMs with evidence for sufficient content validity (any level) and at least low evidence for sufficient internal consistency); (B) PROMs that may have the potential to be recommended, but further validation studies are needed (i.e., PROMs categorized not in A or C); and (C) PROMs that should not be recommended (i.e., PROMs with high quality evidence for an insufficient measurement property). Justifications should be given why a PROM is placed in a certain category, and direction should be given on future validation work, if applicable. We recommend to advise on one most suitable PROM [19]. This recommendation does not only have to be based on the evaluation of the measurement properties, but may also depend on interpretability and feasibility aspects.

#### Step 10. Report the systematic review

In accordance with the PRISMA Statement [22], we recommend to report the following information: (1) results of the literature search and selection of the studies and PROMs, displayed in the PRIMSA flow diagram (including the final number of articles and the final number of PROMs included in the review); (2) characteristics of the included PROMs, such as name of the instruments, constructs being measured, study population for which the PROM was developed, intended context(s) of use, language version of the PROM, number of scales or subscales, number of items, response options, recall period, interpretability aspects, and feasibility aspects; (3) characteristics of the study populations, such as geographical location, language, disease area, target population, sample size, age, gender, setting, and country; (4) methodological quality of each study per measurement property and PROM; (5) a summary of findings (SoF) table per measurement property, including the pooled or summarized results of the measurement properties, its overall rating (i.e., sufficient (+), insufficient (−), inconsistent (+) or indeterminate (?)), and the grading of the quality of evidence (i.e., high, moderate, low, very low). These SoF tables (i.e., one per measurement property) will ultimately be used in providing recommendations for the selection of the most appropriate PROM for a given purpose or a particular context of use. To work towards standardization in outcome measurement (e.g., COS development) and to facilitate meta-analyses, we recommend to advise on one most suitable PROM [19]. This recommendation may also depend on interpretability and feasibility aspects. Examples of tables that can be used for reporting and publishing, can be found in the COSMIN user manual [9]. Note that these tables can be used in the data extraction process throughout the entire review. In addition, we recommend to make the search strategy publicly available, for example on a website or in the (online) supplemental materials to the article at issue.

## Discussion

In the absence of empirical evidence, the COSMIN steering committee developed a methodology for conducting systematic reviews of PROMs that is described in the present guideline. A sequential ten-step procedure for conducting a systematic review of PROMs is recommended. The predefined order of the evaluation of the measurement properties is useful in deciding whether all measurement properties of a PROM should be further evaluated, or whether the PROM can be excluded from further evaluation. Although this guideline was specifically developed for systematic reviews of PROMs, it can also be used as a guidance for reviews of non-PROMs where steps 5–7 should be adapted.

There are a few limitations that we have to acknowledge. The development of the present guideline was not based on a structured process such as the Delphi method or a nominal group technique (i.e., expert panel) and followed by a consensus meeting [42]. We have only applied the methodology in a systematic review on content validity and structural validity [43] and not yet in other reviews. Next, the methods of systematic reviews of PROMs have not yet been fully developed and some aspects need to be further explored. First, we recommend to search in multiple databases. However, the additional value of other databases than PubMed en EMBASE for reviews of PROMs may be limited which has not been systematically evaluated. Second, search filters for finding studies on measurement properties should be developed for other databases besides MEDLINE and EMBASE. Third, methods for statistical pooling of measurement properties are scarce and need to be further developed. Fourth, the sample size requirements that are included in the quality of the evidence table are rules of thumb (further information on sample size requirements can be found in the COSMIN user manual). Fifth, the methods for grading the quality of evidence have not yet been fully worked out. In accordance with the GRADE approach, publication bias is difficult to assess in systematic reviews of PROMs because of a lack of registries for studies on measurement properties. Also, while criteria for downgrading the quality of the evidence now exist, criteria for upgrading (e.g., because of very good measurement properties) have not (yet) been defined. And lastly, future research may be directed towards the evaluation of our methods in terms of reliability or validity.

## Conclusions

This methodological guideline aims to support review authors in conducting systematic reviews of PROMs in a transparent and standardized way. This will contribute to the quality of these reviews and an evidence-based selection of PROMs.

## Abbreviations

AUC: Area under the curve
CFI: Comparative fit index
COMET: Core Outcome Measures in Effectiveness Trials
COSMIN: COnsensus-based Standards for the selection of health Measurement INstruments
CTT: Classical test theory
DIF: Differential item functioning
GRADE: Grades of Recommendation, Assessment, Development and Evaluation
ICC: Intraclass correlation coefficient
IRT: Item response theory
LoA: Limits of agreement
MIC: Minimal important change
PRISMA: Preferred Reporting Items for Systematic Reviews and Meta-Analyses
PROM: Patient-reported outcome measure
SEM: Standard error of measurement
SDC: Smallest detectable change
TLI: Tucker–Lewis index
